# Evaluating the Arterial Stiffness as a Useful Tool in the Management of Obese Children

**DOI:** 10.3390/children10020183

**Published:** 2023-01-18

**Authors:** Monica Simina Mihuta, Dana Stoian, Andreea Borlea, Cristina Mihaela Roi, Oana-Alexandra Velea-Barta, Ioana Mozos, Corina Paul

**Affiliations:** 1 Department of Doctoral Studies, Victor Babes University of Medicine and Pharmacy, 300041 Timisoara, Romania; 2 2nd Department of Internal Medicine, Victor Babes University of Medicine and Pharmacy, 300041 Timisoara, Romania; 3 Center of Molecular Research in Nephrology and Vascular Disease, Faculty of Medicine, Victor Babes University of Medicine and Pharmacy, 300041 Timisoara, Romania; 4 3rd Department of Odontotherapy and Endodontics, Faculty of Dental Medicine, Victor Babes University of Medicine and Pharmacy, 300041 Timisoara, Romania; 5 Department of Functional Sciences—Pathophysiology, Center for Translational Research and Systems Medicine, Victor Babes University of Medicine and Pharmacy, 300173 Timisoara, Romania; 6 Department of Pediatrics, Victor Babes University of Medicine and Pharmacy, 300041 Timisoara, Romania

**Keywords:** cardiovascular dysfunction, pediatric obesity, increased blood pressure, pulse wave analysis, pulse wave velocity

## Abstract

Childhood obesity speeds up the development of arterial stiffness and progressively increases the values of arterial pressure. The purpose of this study is to investigate the value of using pulse wave analysis (PWA) to measure arterial stiffness as a sign of vascular wall impairment in obese children. The research was focused on 60 subjects: 33 obese and 27 normal-weight. Ages ranged from 6 to 18 years old. PWA includes parameters such as pulse wave velocity (PWV), augmentation index (AIx), peripheral and central blood pressure (SBP, DBP, cSBP, cDBP), heart rate, and central pulse pressure (cPP). The device used was a Mobil-O-Graph. Blood parameters were taken from the subject’s medical history, not older than 6 months. A high BMI and a large waist circumference are linked to a high PWV. The levels of LDL-c, triglycerides (TG), non-HDL-c, TG/HDL-c ratio, and total cholesterol-HDL-c ratio significantly correlate to PWV, SBP, and cSBP. Alanine aminotransferase is a reliable predictor of PWV, AIx, SBP, DBP, and cDBP, while aspartate aminotransferase is a significant predictor of AIx, mean arterial pressure (MAP), cSBP, and cPP. 25-OH-Vitamin D negatively correlates with PWV, SBP, and MAP and significantly predicts the MAP. Cortisol and TSH levels are not significant to arterial stiffness in obese children without specific comorbidities and neither is fasting glucose in obese children without impaired glucose tolerance. We conclude that PWA contributes valuable data regarding patients’ vascular health and should be considered a reliable tool in the management of obese children.

## 1. Introduction

Obesity is a silent disease in children. Unhealthy eating and lifestyle behavior are symptoms of various causes that have to do with individual mental health, parental guidance, socio-economic and cultural diversity, access to nutrition, and education [1,2,3]. Globally, 40% of adults have weight excess [4]. In Europe, approximately 60% of men and 50% of women have a variable degree of weight excess [5]. Reports from the World Health Organization and the US Center for Disease Control estimate that childhood obesity’s prevalence ranges between 15% and 20%, depending on regions and cultures [5,6].

The longer the evolution of obesity, the earlier and more likely the debut of complications. One of the most serious complications is the vascular damage associated with chronic inflammation, insulin resistance, and high blood pressure [7]. Cardiovascular illnesses continue to be the main cause of death, representing about 30% of all fatalities. Out of this percent, 85% were caused by a myocardial infarction or a cerebral vascular accident [8]. In adults, it is well-established that the key risk factors for cerebrovascular and coronary artery disease are unhealthy and excessive diets and lifestyles, sedentariness, cigarette smoke, and alcohol abuse [9]. Patients with these behavior patterns will almost always present an excess of adiposity, high blood pressure, high blood sugar and lipids, and a chronic inflammatory syndrome [10].

Obese children present a major risk of becoming obese adults with the above-mentioned disorders. Therefore, one of the main objectives of modern medicine is to decrease the prevalence of pediatric obesity. Prompt detection of vascular disruptions can be one of the means to improve the efforts for achieving this goal. Vascular dysfunction is fueled by processes such as atherosclerosis, arterial stiffness, and high blood pressure. All these processes begin in early childhood, but obesity is an aggravating factor, accelerating all probable complications [11]. The pathological mechanisms behind these developments are based on systemic and vascular inflammation promoted by visceral adiposity [12], which fuels and aggravates dyslipidemia, hyperglycemia, and arterial hypertension [12]. A generous abdominal panniculus is linked to atherosclerotic lesions in young adults [13] and subclinical atherosclerosis in children [14]. The above-mentioned mechanisms also increase arterial stiffness, one of the most reliable markers of endothelial dysfunction. The vascular elasticity is affected primarily by aging [15], but also by the imbalances of nitric oxide and oxygenase caused by inflammation [16], the high blood pressure levels which affect the hemodynamic forces [17], and the insulin-resistance and high low-density lipoprotein levels (LDL-c) [16], which all are the manifestation of obesity, regardless of age [18].

The principal premise of the current research is that, although obese children are not expected to show pathological values for the markers assessing arterial stiffness and blood pressure, they do show increased values in contrast with their normal-weight peers. These values, which are correlated with clinical and blood parameters, may reveal a more comprehensive picture of the cardiovascular and metabolic status of the obese patient and, hence, broach the difficult subject of cardiovascular risk at younger and more malleable ages, when prophylaxis should be more efficient.

The stiffness of the vascular walls can be evaluated by measuring the velocity of the pulse wave (PWV) [19], which reliably predicts cardiovascular incidents in adults [20,21]. In children, most previous studies have linked weight excess and a higher PWV [22,23,24], but there are also some contradicting opinions. Some authors have shown no correlation between the two [25], and some even an inversed correlation [26]. By calculating how quickly the pulse wave travels through the arterial tree, the PWV assesses the vascular elastic characteristics [27,28]. The effect of wave reflection on the waveform of the arterial pressure is essentially a mathematical reconstruction of the aortic pulse wave [29]. The PWV is valuable in clinical practice because it allows timely detection of endothelial dysfunction in patients at risk: a steadily rising PWV causes myocardial remodeling and predicts accurately potential cardiovascular events [30].

The PWA includes another marker which measures the stiffness of the arterial wall, called the index of augmentation (AIx). The AIx is associated with peripheral arterial resistance and, thus, gives valuable information on the elasticity of small arteries [31]. The AIx is an accurate predictor of cardiovascular events and of organ damage in adults [32]. It appears to be less valuable in children, due to its correlation to height: apparently, the smaller the height, the higher the Aix [33]. Multiple studies have detected a lower AIx in children with weight excess that is thought to be linked to their higher activity of the sympathetic neurologic system [33,34,35]. The AIx estimates vascular elasticity by indicating the augmentation component associated to the systole, created by the reflected wave. It represents the ratio of the central to the reflected pulse pressure [36].

The arterial stiffness in individuals with weight excess is linked to high blood pressure levels as well [36]. In children, high blood pressure (SBP, DBP) is usually encountered in adolescents with a long history of obesity, but it can also be seen in children of smaller ages who present severe obesity [37]. The levels of arterial pressure over the 90th percentile in youth are connected with larger PWV values [38]. Moreover, blood pressure is not only linked to arterial stiffness [39], but it is also a reliable predictor of PWV [40].

Multiple techniques, devices, and measurement sites can be used for performing a PWA. The devices are based either on tonometry or oscillometry. Conventionally, especially in adults, two-point estimation methods such as the carotid–femoral PWV or the brachial-ankle PWV are used [41,42]. Single-point brachial oscillometric estimation of the PWV has been validated as well [43] and has a great advantage in children due to the more simplified method of measurement.

The current study investigates the utility of pulse wave analysis (PWA) in measuring the extent of the damage on the arterial wall in children with weight excess. The aim is to show that adding the PWA to our everyday evaluation of such patients is a reliable tool for creating a cardiovascular risk profile that can be useful in patient care. Providing additional scientific data with regard to childhood obesity’s effects on arterial stiffness is an important objective, as it also allows for the implementation of more effective preventive actions in such individuals.

## 2. Materials and Methods

The research took place in the “Pius Brinzeu” Emergency County Hospital from Timisoara, Department of Pediatrics, from January 2022 until June 2022, on 60 children. Ages ranged from 6 to 18 years old.

The recruitment was made from the chronically obese patients who showed up for a medical consult during the study period and who agreed to be part of the study. Only children with stagnating primary obesity were asked to participate (BMI higher than the 95th percentile). We eliminated patients with higher than 5 kg fluctuations in weight over the last 6 months. We also excluded all patients presenting with known contributors to secondary obesity [44], as well as all known factors that enhance arterial stiffness [45].

Children with primary obesity formed the study group. The control group was made up of normal-weight (BMI between the 5th and the 85th percentile), healthy, and age-matched children who were recruited as volunteers to be part of the study.

### 2.1. Physical Exam and Blood Parameters

All subjects were clinically examined: the weight (W, in kg), height (H, in cm), and waist circumference (WC, in cm) were measured, the Body Mass Index (BMI, in kg/m^2^) was calculated, and the puberty development was assessed (Tanner stage). The Mobil-O-Graph device was utilized to determine the peripheral blood pressure values.

The previous six months of each patient’s medical history were used to choose the blood parameters: fasting glucose (mg/dL), high density lipoprotein cholesterol (HDL-c, mg/dL), low density lipoprotein cholesterol (LDL-c, mg/dL), total cholesterol (TC, mg/dL), triglycerides (TG, mg/dL), non-HDL-cholesterol (mg/dL), triglycerides/HDL-c ratio, total cholesterol/HDL-c ratio, cortisol 8 am (ug/dL), thyroid stimulating hormone (TSH, uIU/mL), aspartate aminotransferase (GOT, IU/L), alanine aminotransferase (GPT, IU/L), 25-OH-Vitamin D (ng/mL) and ionized Calcium (mg/dL). These parameters were measured after previous medical consults in our Pediatric Endocrinology Department, following a minimum 12 h time of fasting.

### 2.2. The Evaluation of Arterial Stiffness Using the Mobil-O-Graph

The assessments were done through the Mobil-O-Graph^®^ 24 h ABPM oscillometric device (M26101200, IEM^®^ GmbH, Stolberg, Germany) with the Hypertension Management Software CS (IEM GmbH, Aachen, Germany). It performs a brachial determination of the peripheral BP, followed by a Pulse Wave Analysis which includes the recording of the PWV, index of augmentation (AIx), mean arterial pressure (MAP), heart rate (HR), central pulse pressure (cPP) and the systolic and diastolic central BP (cSBP, cDBP). The device’s one-time measurement function was used. Details of the methodology and specifics of the device have been presented extensively in our previous work [46].

The manufacturer recommends the device for pediatric use, in children over three years old. Our experience confirms its operational reliability as well [46]. The software is enhanced with the AHA limits for BP for children. The IEM Mobil-O-Graph device was chosen because, in addition to being a validated device [47], its use is facile, reproducible, and has very good acceptability in children. Multiple studies confirm that the oscillometric devices acquire accurate measurements even in children [47,48,49].

The patients were instructed to avoid smoking for at least 4 h before to the examination, avoid consuming caffeinated beverages, and go to bed at 10 p.m. at the latest the night before our meeting. Each subject’s arm circumference was determined, and the appropriate cuff size was selected (extra small: 5.5–7.8 inches; small: 7.8–9.4 inches; medium: 9.4–12.6 inches; large: 12.6–14.9 inches). We took the measurement after a 10-min period of supine rest. On the subject’s naked upper arm, the cuff was placed with the pressure tube facing up and the artery sign on the brachial artery. The patient must remain still and silent throughout the measuring process [46].

The Mobil-o-Graph performs two separate measurements: Firstly, the measurement of the peripheral BP values, followed by a thirty-second intermission; secondly, the PWA, the central BP, and pulse pressure measurement; lastly, the software analyzes the measurement’s accuracy and advises on accepting the data or repeating the measurement. Each patient underwent one measurement. If the measurement’s accuracy was poor, we retook it after a five-min break [46].

### 2.3. Statistical Analysis

Data collection was carried out using Microsoft Excel and statistical analysis was conducted using MedCalc Statistical Software version 20.111 (MedCalc Soft-ware Ltd., Ostend, Belgium).

The 60 subjects formed two groups, according to the inclusion criteria: an obese group (*n* = 33) and a control group (*n* = 27, normal-weight children). Multiple subgroups were needed for statistical analysis based on sex, age, and puberty development. These groups were analyzed focusing on the parameters acquired through the oscillometer, which we called “the PWA parameters” in the Results section, for simplification: PWV (m/s), AI %, SBP (mmHg), DBP (mmHg), MAP (mmHg), HR (b/min), cSBP (mmHg), cDBP (mmHg), and cPP (mmHg).

The Shapiro–Wilk test divided the approach of the statistical analysis, splitting the data into normal and non-normal with regard to its distribution. Means, the Student’s *t*-test, and Pearson’s correlations were used for the first situation and medians; the Mann-Whitney test and Spearman’s correlations for the latter. The *p*-value under 0.05 was used for significance. Multiple analyses of the same variables were conducted (3 groups compared two-by-two). In order to maintain a significance level of less than 0.05, one-way ANOVA post-hoc tests were run and Bonferroni corrections were made. The stepwise approach of multivariable regression analysis was used in MedCalc.

### 2.4. Ethics Approvals

The study respects the Helsinki Declaration and has received approval from the University of Medicine and Pharmacy Victor Babes Timisoara’s Ethics Committee for Scientific Research (No. 03/19.01.2021). We acquired the informed consent of all legal guardians and 18-year-old patients and the verbal consent of all minor patients.

## 3. Results

### 3.1. General Description

Sixty subjects between the ages of 6 and 18 (mean age 11.71 years, SD = 3.38) were involved in the study. Based on their BMI, two groups of study were created, one of obese subjects (15 girls and 18 boys) and one of controls (14 girls and 13 boys). Table 1 depicts the differences between the analyzed markers in the study groups.

### 3.2. The BMI and Arterial Stiffness Parameters

**Figure 1:**
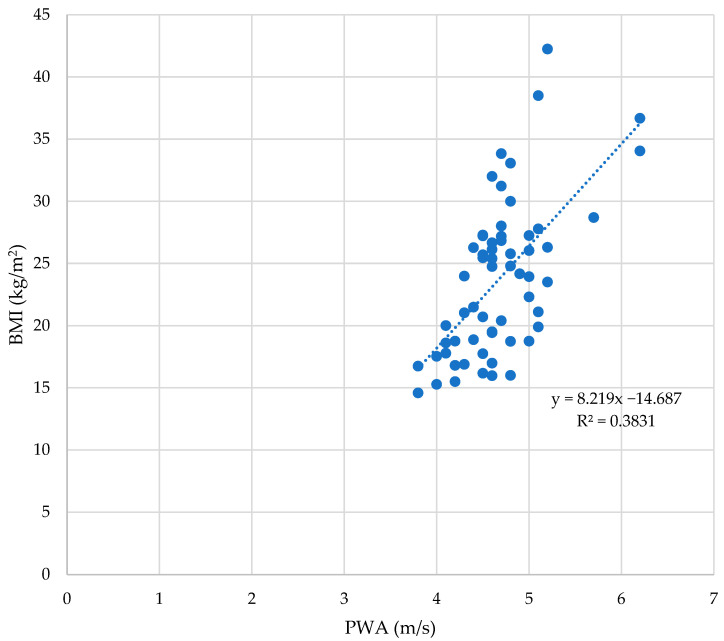
The PWV correlates to BMI values significantly in all subjects (ρ = 0.59, *p* < 0.0001).

The majority of PWA markers significantly correlated with BMI as can be seen in Table 2 as well as in Figure 1.

### 3.3. Sex and Arterial Stiffness Parameters

Fifteen obese girls (45.45%) and 18 obese boys (54.54%), as well as 14 girls (51.85%) and 13 boys (48.14%) acting as controls were evaluated. Table 3 displays the comparisons between the examined data for each subgroup.

The comparison of same-sex participants from the two groups revealed significant differences in arterial stifness parameters (Table 4).

### 3.4. Age and Arterial Stiffness Parameters

Under twelve years old, twelve to fifteen years old, and over sixteen years old comprised the three age subgroups for each study group. Following the Bonferroni adjustment, the one-way ANOVA tests failed to find any differences between the three subgroups for obese participants (PWV (*p* = 0.28), AIx (*p* = 0.81), SBP (*p* = 0.34), DBP (*p* = 0.06), MAP (*p* = 0.15), HR (*p* = 0.42), cSBP (*p* = 0.34), cDBP (*p* = 0.07), and cPP (*p* = 0.64)), or controls ((*p* = 0.81), DBP (*p *= 0.64), MAP (*p* = 0.056), HR (*p* = 0.82), cSBP (*p* = 0.64), cDBP (*p* = 0.19), and cPP *(p* = 0.27)). Table 5 demonstrates that post-pubertal subjects had higher values for the majority of the arterial stiffness parameters tested in the obese group.

Table 6 shows significant differences between the same-age subjects belonging to the two groups for PWV (*p* = 0.03), HR (*p* = 0.03), cSBP (*p* = 0.009), and cPP (*p* = 0.01) in obese subjects younger than 12, and PWV (*p* = 0.004), SBP (*p* = 0.02), cSBP (*p* = 0.002), and cDBP (*p* = 0.005) in obese subjects aged 12–15.

Because the normal weight group only had four subjects and the obese group six, the analysis of the subjects ≥16-year-old is statistically inaccurate.

### 3.5. Tanner Stages and Arterial Stiffness Parameters

According to Tanner’s stages of puberty, the research groups were split into three subgroups that included stage I, II and III, and IV and V. The ANOVA did not reveal considerable variations in arterial stiffness parameters between the three categories after applying the Bonferroni adjustment. Most parameters showed higher scores in Tanner IV and V patients (Table 7).

In obese subjects, for Tanner I children, the analysis revealed increased scores for PWV (*p =* 0.017) and cSBP (*p* = 0.009) compared to their controls; for Tanner II and III, higher PWV (*p* = 0.03) and cSBP (*p* = 0.027) values; and for Tanner IV and V, higher cSBP (*p* = 0.014) and cDBP (*p* = 0.0095) (see Table 8).

### 3.6. Waist Circumference and Arterial Stiffness Parameters

For subjects who were obese, the average waist size was 98.3 cm (Table 1). The research detected positive correlations between obese subjects’ waist circumferences and the markers of arterial stiffness (PWV and BP values, Table 9). For controls, the average waist size was 63.3 cm (Table 1). Positive correlations were observed for PWV, SBP, MAP, and cSBP for controls also (Table 9). The WC of obese subjects was significantly higher than of controls (*p* < 0.01).

**Figure 2:**
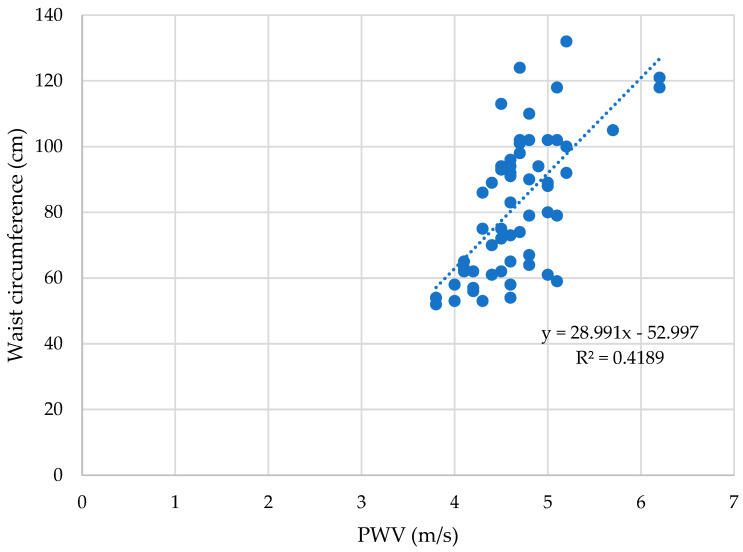
The WC correlates with the pulse wave velocity significantly(ρ = 0.63, *p* < 0.01).

When evaluating all subjects, the WC displayed a strong positive correlation with the PWV (ρ = 0.63, *p* < 0.01); Figure 2.

**Figure 3:**
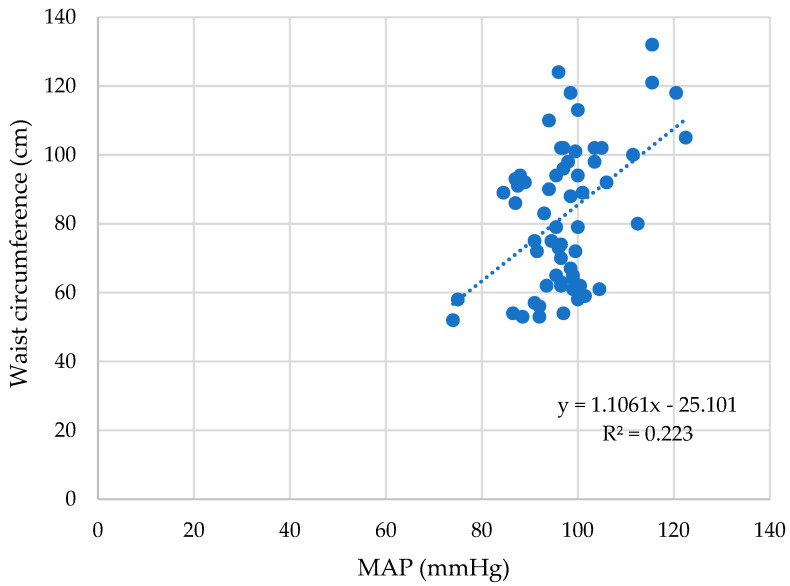
The waist circumference correlates with the MAP significantly (ρ = 0.38, *p* < 0.01).

The waist circumference has a strong correlation with MAP values as well (ρ = 0.38, *p* < 0.01), as shown in Figure 3.

### 3.7. Blood Parameters and Arterial Stiffness Parameters

The analysis of arterial stiffness parameters in relation to blood parameters, in the obese group, showed significant correlations as depicted in Table 10. HDL-c and total cholesterol analysis showed *p*-values close to the significance and the overall trend of the mean values were the ones expected.

The analysis of PWA parameters in relation to blood parameters, in the normal-weight group, showed significant correlations as well, as depicted in Table 11. PWV, cSBP, cDBP mmHg were significantly negatively correlated to HDL-c.

### 3.8. Multilinear Regression Model

The complete lot was subjected to a regression analysis in order to find which parameters of the arterial stiffness could be independently predicted by blood parameters. The PWA parameters were dependent variables, each in turn. Table 12 depicts the results of the regression. The independent variables that were not mentioned in Table 12 were not detected as significant predictors.

## 4. Discussion

The research is focused on the parameters measured by the Mobil-O-Graph device and how they correlate to the clinical and blood parameters of the subjects. The results can help clinicians in their everyday activity to create a more accurate risk profile for their obese pediatric patients, to be more efficient in providing advice regarding treatment, lifestyle, and diet changes [50], and ultimately to lower their risk of becoming cardiovascular- and metabolic-impaired adults. It is worth noting right from the start that both the peripheral and central SBP may not be very accurate in such devices, with some authors suggesting that the values could be overestimated [51]. Our work is unique in that it not only examines arterial stiffness markers collected by the oscillometer in relation to childhood obesity, but it also shows their correlations to parameters beyond the clinical examination ones. The study mainly shows that the levels of blood lipids, transaminase, and 25-OH-Vitamin D significantly correlate to the surrogate markers of arterial stiffness. In other words, the most important metabolic parameters used in the daily evaluation of obese pediatric patients are correlated with non-invasive measurements of vascular stiffness. These results confirm the premise that the progression of stiffness in the vascular walls is an issue that affects obese children earlier than their normal-weight peers. Hence, the study advances pertinent information and is a significant step toward realizing the goal of relying on the PWA in routine clinical activity.

Opinion on whether arterial stiffness markers such as PWV, AIx, and even blood pressure values are indeed affected in obese children is divided. Although these are well-known indicators of vascular elasticity degradation in obese adults, more research is required to determine their effects on vascular elasticity in children. Although many studies including our own have shown increased values of PWV and of peripheral and central BP in children with weight excess [18,20,22,23,46], a very large study on 6816 children aged 3 to 18 years by Jakab et al. showed contrary results [35].

The clinical evaluation of the two groups of study included weight, height, BMI, sex, age, Tanner stage, and waist circumference. The blood parameters analysis included: HDL-c, LDL-c, TC, TG, non-HDL-, TG/HDL-c ratio, TC/HDL-c ratio, fasting glucose, cortisol 8 am, TSH, GOT, GPT, 25-OH-Vitamin D, and ionized Calcium. These are parameters normally used in pediatrics or endocrinology practices which, associated with measurements such as the PWA parameters, may help paint a clearer picture of the patient’s cardiometabolic status and cardiovascular risk.

Studies show that excess adipose tissue is associated with elevated arterial stiffness, as well as elevated blood pressure levels [23,24], which raises the value of its surrogate measures such as PWV [19,20]. The results support the data suggesting that the PWV, SBP, MAP, cSBP, cDBP, and cPP are linked to weight and BMI. DBP significantly correlated to weight and height, but not BMI. The BMI is a reliable marker of excess adipose tissue; hence, its correlation to PWV (ρ = 0.59, *p* < 0.0001, Figure 1) proves that adiposity strongly correlates to a rise in arterial stiffness. For adults, height has an inverse effect on vascular parameters [52,53,54]. This is not the case for children whose growth is still ongoing [54]. Moreover, both weight and height are significantly correlated to most PWA parameters; hence we can affirm that the correlations between the BMI and the PWA parameters are accurate. Some studies suggest that central BP values acquired through tonometry and oscillometry devices may be underestimated [55,56]; however, normal ranges of the central BP values in children are not entirely supported. This study showed a strong positive link between the BMI and cSBP, and cDBP, respectively, meaning that excess weight is associated with higher central BP values. Harbin et al. showed that the central aortic BP among individuals with morbid obesity is significantly higher (over 120% of the 95th percentile for BMI) in a study on 348 patients aged 8–18 in which measurements were acquired through a tonometry device. Moreover, they showed that the patients’ central BP is linked to their BMI, but not their body fat mass % [55].

It appears that sex has little to no impact on the vascular elasticity of obese children. Harbin et al. showed no variations between sexes concerning central BP values [55]. In an age-matched study, pre-pubescent females presented elevated levels of the velocity of the pulse wave and central BP compared to same-age males, while in teenagers who have completed their puberty, males presented higher values for PWV than females [57]. Differences in adult male and female vascular health are linked to their hormonal profiles, as sex hormone receptors are expressed in vessels [58]. Estrogen and progesterone are historically established as cardio-protective hormones [59,60]. Testosterone is positively involved in arterial reactivity [61] and compliance [62]. It also negatively predicts arterial stiffness in men [63]. This study supports the data that in children, sex does not cause significant differences in endothelial health. However, we did discover that obese girls had substantially higher index of augmentation (*p* = 0.038) and heart rate levels than obese boys in this study (*p* = 0.039). Obesity considerably raises the scores of PWV, SBP, cSBP, and cDBP when evaluating PWA parameters within each sex. The fact that obese girls present increased markers for arterial stiffness compared to normal-weight girls, and that the same is valid for boys, is another argument that excess weight increases arterial stiffness, including peripheral and central BP values.

Age has an uncertain and difficult-to-measure impact on vascular stiffness in the pediatric and young-adult population. The main causes for vascular damage due to age are connected to the loss of collagen and elastin within the tissue of the vessels [64]. In obese adolescents, the main pathological mechanisms behind endothelial damage are processes such as inflammation, dyslipidemia, insulin resistance, and high blood pressure, which may exert stronger effects on vessels than the age-related loss of collagen and elastin [27]. PWV and blood pressure in adults both correlate with age; however, the data are more evenly distributed when the patients are younger [65,66]. The analysis did not observe any significant variations between the three subdivided groups (<12, 12 to 15, and ≥16 years old), regardless of the BMI. Small obese children under 12 displayed increased scores for PWV, HR, cSBP, and cPP compared to their normal-weight age-matched controls, while obese children between the ages 12 and 15 showed significantly higher PWV, SBP, HR, cSBP, and cDBP than their controls. Subjects over 16 years old were too few to perform a significant statistical comparison.

Puberty itself is not damaging to arterial elasticity, nor is it associated with increased velocity of the pulse wave or the index of augmentation in obese children [67]. The expected differences between pre-pubertal and post-pubertal children could be caused by excess androgens in girls, as is the case in many obese girls [68]. Normal testosterone levels in boys are beneficial to their arterial health [63]. The analysis did not reveal any variations between the groups subdivided by puberty stages. Overall mean values were higher in post-pubertal children, but statistical significance was not met after the Bonferroni corrections. Obesity does have an effect on the central blood pressure of same puberty stage children, as each Tanner stage presented more elevated central BP levels than their same puberty stage controls.

Abdominal adiposity represents a reliable marker of metabolic disruption and cardiovascular events [69], an essential part of MetS and resistance to insulin [70]. Not only is waist circumference associated with subclinical atherosclerosis, but it also predicts the thickness of the carotid intima-media [14,71]. This study detected positive correlations between the waist circumference of obese children and all of the arterial stiffness markers. 

The analysis of blood parameters represents the particularity of the present study; as previously discussed, our previous work revealed similar results for clinical parameters [46].

HDL-c is the anti-inflammatory component of the lipid panel, its main role being to transport excess cholesterol to the hepatic cells from the arterial walls [72]. The metabolic syndrome, especially in sedentary children, is characterized by lower HDL-c levels, increasing the predisposition for increased risk of atherosclerosis progression and arterial stiffness [73]. In the study, multiple regression revealed that HDL-c is a significant predictor of AIx and of DBP. However, although the values of HDL-c were lower in obese subjects compared to controls, significance was not met for any of the PWA markers. For controls, HDL-c showed negative significant correlations to PWV, Aix, cSBP, and cDBP.

LDL-c is a major player in atherosclerotic plaque formation, especially when it is assocciated with low HDL-c [73]. When a child presents both these alterations, their cardiovascular long-term prognosis is negatively affected [73,74]. The study detected significant correlations between LDL-c and PWV, SBP, and cSBP, in obese children. Not only is the LDL-c correlated to these important vascular markers, but the regression showed that its value is also a predictor of PWV, SBP, cSBP, and cDBP.

Tryglicerides, another component of the metabolic syndrome, reflects the individual proclivity to an unhealthy lifestyle, its values being under the direct influence of each individual’s diet [75]. Because the TG levels are so influenced by eating habits, there is significant personal variability, which makes TG a less stable parameter than HDL-c [75]. However, multiple studies have shown that TG are important in assessing the cardiovascular health of patients at risk [75]. This analysis has detected significant correlations with PWV, SBP, MAP, and cSBP in the obese group. Correlations with SBP and cSBP were also revealed in the control group. Moreover, the multiple regression showed that TG represents a significant predictor of cPP.

In adult populations, the values of these blood lipids are clearly linked to vascular damage. The work of Wang et al. involving 909 adults revealed, after accounting for cardiovascular risks, that HDL levels are negatively correlated with PWV, while TG, LDL-c, and total cholesterol levels are positively correlated with PWV. The study also suggests that the otherwise healthy population displaying significant risk factors for arterial elasticity loss should take into account lipid-lowering therapy [76]. Therefore, maintaining a normal weight, diet, and physical activity since childhood is an efficient prevention of vascular damage in adult life, in many cases even avoiding the need for lipid-lowering therapy. 

Studies show the ratio between TG and HDL-c may be a more consistent and accurate indicator for cardiovascular, metabolic and dysglicemia risk [77,78]. This study showed its correlations with PWV, SBP, MAP, and cSBP in the obese group. For controls, correlations were established with AIx, SBP, MAP, and cDBP. Furthermore, the regression showed that the TG/HDL-c ratio reliably predicts the values of cPP and heart rate.

Non-HDL-c is at least as valuable in assessing cardiovascular risk as is LDL-c, being correlated to the risk of atherosclerotic progression and long-term cardiovascular events [79]. In both the obese and the control group, the analysis detected correlations between non-HDL-c and PWV, SBP, and cSBP.

The ratio between TC and HDL-c represents an independent predictor of ischemic coronary events in adults. Its power as a predictor grows when individuals with high TC/HDL-c ratio also present large waist circumferences, insulin resistance, and dyslipidemia [80]. The analysis showed significant correlations with PWV, SBP, and cSBP for the obese subjects. In the control group, correlations with the peripheral and central BP, but MAP as well, were detected. Moreover, the TC/HDL-c ratio sgnificantly predicts MAP and cDBP.

High fasting glucose levels represent one of the earliest signs of glucose–insulin metabolism impairment in adults [81]. However, in children with obesity, due to the individually variable levels of insulin resistance, glucose levels can present fluctuating levels and, thus, in our experience as well as in others [82], fasting glucose may not be the most accurate parameter. Moreover, the literature reports significant differences between the prevalences of impaired fasting glucose (IFG) in obese children in different geographical and ethnical populations: for instance, Swedish obese children presented significantly higher prevalenges of IFG than German and Polish obese children [83,84]. In this study, although the obese presented significantly higher mean fasting glucose values than the normal-weight (*p* = 0.01), only 6% of the obese presented a fasting glucose >110 mg/dL. Moreover, no correlations were detected in our analysis between fasting glucose and PWA parameters. These findings are in line with the above-mentioned observations. A far more accurate marker for insulin resistance is the HOMA-IR score, which in adolescents with weight excess is known to be a trustworthy proxy for estimating the resistance to insulin activity, powerfully predicting the risk of developing diabetes mellitus type 2 [85]. An important longitudinal study on preadolescent children showed that being overweight or obese is the main cause of insulin resistance for that age group as well [86]. Hence, for all age groups, titration of serum insulin and calculation of HOMA-IR is far more valuable than evaluating fasting glucose. However, titration of insulin is not a standard practice and is commonly not covered by standard insurance in many countries. This could be an impediment in many clinical practices. The glucose oral tolerance test is also a trustworthy test for detecting glucose alterations [87], but unfortunately, it is time-consuming, may raise difficulties for the setting of ambulatory clinical practices and, last but not least, may be less reproducible than other methods [88]. Hemoglobin A1c is not an ideal marker, as its sensitivity lacks in identifying glucose metabolism impairments for either fasting glucose or glucose tolerance. Hence, it has no obvious usefulness in detecting early or subtle anomalies in glucose excursions [88]. Hence, future research should use HOMA-IR or the oral glucose tolerance test as markers of glucose metabolism dysfunction in obese children in order to improve comprehension of how it affects the markers of vascular stiffness.

The most frequent liver issue associated with childhood obesity is non-alcoholic fatty liver disease, affecting almost 80% of these patients in various degrees of severity [89]. It is a disorder associated with higher than normal GPT ± GOT. Several studies put patients with NAFLD at a higher risk of developing cardiovascular disorders, objectified in a higher arterial stiffness and progressive subclinical atherosclerosis [90]. For GPT, the study showed significant correlations with PWV, SBP, DBP, MAP, and cDBP. In the control group, correlations with PWV, AIx, DBP, and HR were detected. Moreover, the multilinear regression showed that GPT confidently predicts PWV, AIx, SBP, DBP, and cDBP. For GOT, we detected significant correlations with PWV, SBP, DBP, MAP, cSBP, and cDBP in the obese group. For controls, GOT was correlated only to cPP. GOT is also an independent predictor of AIx, MAP, cSBP, and cPP. A study on obese adolescents also linked higher liver enzymes to both increased arterial stiffness and a higher carotid intima-media thickness [91]. Hence, we can say that indeed, both GPT and GOT are linked to cardiovascular health and both are significant to the progression of arterial stiffness in children.

Cortisol oversecretion is linked to cardiovascular conditions and thus, represents a significant contributor to morbidity and even death in Cushing’s disease [92]. Obese children can present higher levels of cortisol as a result of their metabolic imbalance, but levels of serum cortisol are much lower than in Cushing’s disease [93]. Excess cortisol is involved in arterial hypertension, insulin resistance, dyslipidemia, impaired glucose tolerance, and weight gain. This study analyzed the subjects from the perspective of obesity-caused cortisol excess and not the reverse, but did not find a link bewteen serum cortisol titers and PWA parameters. These results can be attributed to the fact that most of the subjects presented serum cortisol levels within the normal limits.

The thyroid-stimulating hormone is linked to arterial stiffness, meaning higher values of the PWV, in overt or subclinical hypothyroidisms [94,95]. Weight excess in children can cause the benign rise in TSH [96]. No correlations were found in our study between the TSH levels and the PWA parameters, neither for obese children nor for controls, probably due to the fact that TSH serum titers were not very elevated compared to the normal ranges in the children included in the study.

25-OH-Vitamin D is involved in multiple intricate systems of the human organism, with great importance in phosphate–calcium metabolism, immune modulation, and vascular protection. Its involvement in the endothelial wall is controlled by the relationship of the endothelial cell vitamin D receptors and ligand vitamin D [97]. It also regulates the renin–angiotensin–aldosterone axis and inhibits the proliferation of vascular smooth muscle [98]. Obese children commonly display low to very low titers of 25-OH-Vitamin D in the absence of substitution and/or appropriate sun exposure [99,100]. In this research, 25-OH-Vitamin D negatively correlates with PWV, SBP, and MAP. No correlations were detected in normal-weight children. According to the multilinear regression, 25-OH-Vitamin D predicts the value of MAP. With regard to other blood parameters, Censani et al. showed that in addition to being deficient in obese children, 25-OH-Vitamin D is also associated with an increase in several atherogenic lipids [101]. A recent clinical trial found that, although corrected vitamin D deficiency had no effect on the markers of vascular elasticity or function, generalized inflamation or blood lipids, it did lower the blood pressure and improve insulin sensitivity in children with weight excess [102]. Hence, a low 25-OH-Vitamin D serum level represents a modifiable cardiovascular and metabolic risk factor and obese children should benefit from correct suplementation in order to reduce risk. In obese children with Vitamin D deficiency and pro-inflammatory status, serum ionized Calcium (iCa) can either be high, due to reactive hyperparathyroidism, or low, due to a rich but unbalanced diet [103]. Progressive vascular calcifications appear in metabolically impaired individuals on behalf of the oxidative stress and the signals that are controlled by it in endothelial smooth-muscle cells [104]. The obese subjects presented negative correlations for their serum iCa levels with PWV, peripheric and central BP, and MAP in our observations. In the control group, significant positive correlations were detected with the same markers, except for PWV. Most studies on adults linking iCa levels to arterial stiffness refer to patients with hypoparathyroidism, in which case low iCa is associated with high PWV; or to patients with chronic kidney disease which is often associated to secondary hyperparathyroidsm, in which case high phosphorus and low iCa correlates with high PWV and BP levels [105]. In patients undergoing haemodialysis, high iCa is correlated to high PWV [106]. This situation is studied in children on dialysis as well, in which case the balance of calcium–phosphorus represented by the levels of PTH and an appropriate vitamin D dosage are important and distinct indicators of vascular calcifications and the degradation of the arterial walls [107]. Unfortunately, studies on iCa and vascular health in obese children are lacking. This study is unique to our knowledge in showing a negative correlation between serum iCa and PWV and BP levels. However, one does not expect to find extreme values of iCa in obese, but otherwise healthy, children. Hence, in order to reduce risk, both vitamin D and iCa should be measured, corrected, and monitored.

The primary benefit of this research is that the results managed to significantly support the main starting hypothesis that obese children present increased arterial stiffness and higher blood pressure values. Another strength is that the study succeeded in revealing the associations between the non-invasive markers of arterial stiffness and clinical parameters, and metabolic blood parameters, respectively. The limitations of this study include failing to perform ultrasonography measurements of the flow-mediated dilation, which is regarded as a standard for the early identification of damage in the arterial walls [108], as well as liver ultrasonography [109] or liver elastography measurements for assessing the possible non-alcoholic fatty liver disorder development [110]. Moreover, although having performed a pre-statistical estimation of the sample size before starting the study, we believe that in some cases, the groups of study could have been larger (for instance, the number of subjects ≥16 years old was too small in some situations). However, our research was statistically significant and consistent with prior works. Hence, notwithstanding the relatively limited sample, this work offers valuable data on arterial stiffness in obese children and should foster future more extensive research expanding the sample sizes and the number of analyzed metabolic, endothelial, and imagistic markers, especially regarding the glucid metabolism (oral glucose tolerance tests, insulin levels, and HOMA-IR) and liver function.

## 5. Conclusions

Childhood obesity promotes the loss of elasticity of large vessels by progressively aggravating arterial stiffness and increasing the values of peripheral and central blood pressure. A high BMI and an increased waist circumference are associated with a high PWV.

Increased levels of LDL-c, triglycerides, non-HDL-c, as well as TG/HDL-c, TC/-HDL-c ratios, transaminase levels, and low titers of 25-OH-Vitamin D are significant to the progression of arterial stiffness in obese children. Cortisol and TSH levels are not significant to arterial stiffness in obese children without specific endocrine comorbidities and neither is fasting glucose in obese children without impaired glucose tolerance.

We conclude that PWA contributes valuable data regarding patients’ vascular health, helping clinicians not only to evaluate and monitor patients at risk more accurately, but also to be more effective in their combat against obesity. Therefore, we believe that PWA should be considered a reliable instrument in the everyday evaluation of obese children.

Future scientific endeavors will be required to better integrate the non-invasive markers of arterial stiffness with those of early endothelial dysfunction (flow-mediated dilation) and of subclinical atherosclerosis (carotid intima-media thickness) in obese children.

## Figures and Tables

**Table 1:** Differences in obese vs. normal-weight subjects.

	Obese Group		Control Group		
	Mean/Median	SD	Mean/Median	SD	*p*-Value
BMI	26.8	4.61	18.21	2.04	**<0.01**
WC	98.32	13.82	63.37	8.1	**<0.01**
PWV	4.7	0.45	4.4	0.3	**<0.01**
AIx	24.34	10.92	22.94	10.42	0.61
SBP	122.3	11.38	115	8.6	**<0.01**
DBP	75.91	10.21	78	7.95	0.88
MAP	98	9.55	96.5	7.82	0.08
cSBP	113	11.02	100.71	10.03	**<0.01**
cDBP	77.42	9.91	69.11	8.62	**<0.01**
cPP	37.89	10.53	32	5.7	**0.01**
HR	88.72	11.09	83.72	6.32	**0.04**
Fasting glucose	88.68	14.47	80.49	10.78	**0.01**
HDL-c	41	8.42	39	10.6	0.34
LDL-c	131.81	36.02	93.31	34.55	**<0.01**
Total cholesterol	187.22	32	160.21	27.89	**<0.01**
Triglycerides	112	53.71	80	49.1	0.14
non-HDL-c	146.5	35.32	117.18	29.2	**<0.01**
Triglycerides/HDL-c ratio	2.9	1.78	2	1.3	0.09
Total cholesterol/HDL-c ratio	4.3	1.55	4	1.05	**<0.01**
Cortisol	18.4	4.1.1	17	2.2	0.17
TSH	4.2	1.72	3.5	0.9	0.06
GOT	34.4	14.03	21	7.8	**<0.01**
GPT	32	18.2	24	9.3	**<0.01**
25-OH-Vitamin D	28.5	14.11	24	8.9	0.1
Ionized Calcium	4	0.3	4.1	0.22	0.83

Statistically significant results are indicated in bold.

**Table 2:** PWA parameters in correlation to BMI, in all children, using Spearman’s correlation (ρ) (N = 60).

	PWV	AIx	SBP	DBP	MAP	HR	cSBP	cDBP	cPP
BMI	**ρ = 0.59**	ρ = 0.18	**ρ = 0.53**	ρ = 0.14	**ρ = 0.36**	ρ = 0.18	**ρ = 0.65**	**ρ = 0.45**	**ρ = 0.36**
*p*-value	**<0.01**	0.15	**<0.01**	0.26	**<0.01**	0.16	**<0.01**	**<0.01**	**<0.01**
W	**ρ = 0.68**	**ρ = 0.29**	**ρ = 0.64**	**ρ = 0.36**	**ρ = 0.48**	ρ = 0.16	**ρ = 0.64**	**ρ = 0.50**	**ρ = 0.38**
*p*-value	**<0.01**	**0.03**	**<0.01**	**0.006**	**<0.01**	0.21	**<0.01**	**<0.01**	**<0.01**
H	**ρ = 0.50**	**ρ = 0.29**	**ρ = 0.49**	**ρ = 0.42**	**ρ = 0.52**	ρ = −0.02	**ρ = 0.46**	**ρ = 0.52**	ρ = 0.15
*p*-value	**<0.01**	**0.024**	**<0.01**	**<0.01**	**<0.01**	0.87	**<0.01**	**<0.01**	0.24

Statistically significant results are indicated in bold.

**Table 3:** Comparison between sexes regarding the arterial stiffness parameters.

	Sex	PWV	AIx	SBP	DBP	MAP	HR	cSBP	cDBP	cPP
Obese	Boys	4.8	19.5	120.5	75.5	98.05	85.5	113.7	76.3	41
	Girls	4.79	38.6	118	75	97	93	113.8	78	36
	*p*-value	0.56	**0.04**	0.94	0.97	0.66	**0.04**	0.67	0.77	0.35
N-weight	Boys	4.4	23	114.4	77.2	95.8	83.7	100.6	68.8	31
	Girls	4.41	22.7	114.5	77	96.5	83.7	100.5	69.4	30.5
	*p*-value	0.67	0.94	0.66	0.39	0.64	0.97	0.27	0.86	0.17

Statistically significant results are indicated in bold.

**Table 4:** PWA comparison between the same sexes (obese vs. N-weight).

	PWV	AIx	SBP	DBP	MAP	HR	cSBP	cDBP	cPP
Obese girls	4.79	38.6	118	75	97	93	113.8	78	36
N-weight girls	4.41	22.7	114.5	77	96.5	83.7	100.5	69.4	30.5
*p*-value	**<0.01**	**0.01**	**0.03**	0.63	0.41	**0.02**	**<0.01**	**0.02**	0.11
Obese boys	4.8	19.5	120.5	75.5	98.05	85.5	113.7	76.3	41
N-weight boys	4.4	23	114.4	77.2	95.8	83.7	100.6	68.8	31
*p*-value	**0.01**	0.34	0.07	0.63	0.49	0.49	**<0.01**	**0.04**	0.059

Statistically significant results are indicated in bold.

**Table 5:** Overall scores of the arterial stiffness markers in obese patients of the 3 age groups.

	*n*	PWV	AIx	SBP	DBP	MAP	HR	cSBP	cDBP	cPP
<12 years	12	4.6	24.8	116	70.7	94.7	92	108	72.8	38.4
12–15 years	15	4.75	23.5	121.6	77.9	99.7	87.9	113.9	79	36.5
≥16 years	6	5.1	25.1	128.7	80.7	104.7	84.5	122	82.1	39.8

**Table 6:** Arterial stiffness parameters comparison in subjects of the same ages: obese vs. N-weight.

		*n*	PWV	AIx	SBP	DBP	MAP	HR	cSBP	cDBP	cPP
<12 years	Obese	12	4.6	24.8	116	68	94.7	92	108	72.8	41
	N-weight	13	4.2	22.7	110	75	92	84	102	67.6	30
	*p*-value		**0.03**	0.64	0.07	0.17	0.91	**0.03**	**<0.01**	0.2	**0.01**
12–15 years	Obese	15	4.75	23.5	121.6	78	99.7	87.9	113.9	79	40
	N-weight	10	4.5	21.6	115.8	78	96.8	82.1	103	68.9	31
	*p*-value		**<0.01**	0.68	**0.02**	0.91	0.24	0.07	**<0.01**	**<0.01**	0.27

Statistically significant results are indicated in bold.

**Table 7:** Overall scores of the arterial stiffness parameters in obese patients in the 3 puberty development stages.

Puberty Stage	*n*	PWV	AIx	SBP	DBP	MAP	HR	cSBP	cDBP	cPP
I	8	4.5	22.3	111.5	69.5	93.2	91.7	106.5	75.1	40
II, III	13	4.8	23.5	121.7	73	97.3	88.7	114.3	74.6	39.2
IV, V	12	4.85	26.5	122	80	99	86.6	118.4	82	37

**Table 8:** Arterial stiffness parameters in subjects with the same Tanner Stages: obese vs. N-weight.

		*n*	PWV	AIx	SBP	DBP	MAP	HR	cSBP	cDBP	cPP
Tanner I	Obese	8	4.5	22.3	111.5	69.5	93.2	91.7	106.5	75.1	40
	N-weight	10	4.15	20.1	109.5	75	92	84.1	92	65.1	27.5
	*p*-value		**0.01**	0.65	0.15	0.53	0.59	0.12	**<0.01**	0.05	0.08
Tanner II, III	Obese	13	4.8	23.5	121.7	75	97.3	88.7	116	74.6	39.2
	N-weight	11	4.5	24.5	116.5	81	97.5	82.7	106	72.8	34.9
	*p*-value		**0.03**	0.83	0.06	0.14	0.96	0.05	**0.02**	0.6	0.21
Tanner IV, V	Obese	12	4.85	26.5	122	80	99	86.6	118.4	82	37
	N-weight	6	4.65	24.6	117	76	96.2	85	103.1	69.1	30
	*p*-value		0.18	0.73	0.23	0.74	0.26	0.77	**0.01**	**<0.01**	0.45

Statistically significant results are indicated in bold.

**Table 9:** Waist circumference and arterial stiffness parameters (Pearson’s r and Spearman’s ρ).

	PWV	AIx	SBP	DBP	MAP	HR	cSBP	cDBP	cPP
Obese	**ρ = 0.53**	r = 0.24	**ρ = 0.49**	**r = 0.46**	**r = 0.53**	r = −0.08	**ρ = 0.51**	**r = 0.42**	r = 0.18
*p*-value	**<0.01**	0.17	**<0.01**	**<0.01**	**<0.01**	0.64	**<0.01**	**0.015**	0.31
Controls	**r = 0.57**	r = 0.13	**r = 0.57**	ρ = 0.31	**ρ = 0.42**	r = −0.17	**r = 0.43**	r = 0.21	ρ = 0.28
*p*-value	**<0.01**	0.49	**<0.01**	0.10	**0.02**	0.36	**<0.01**	0.28	0.15

Statistically significant results are indicated in bold.

**Table 10:** Correlations between arterial stiffness parameters and blood parameters in obese children.

Correlations for Obese Patients	PWV	AIx	SBP	DBP	MAP	HR	cSBP	cDBP	cPP
HDL-c	ρ = −0.32	ρ = −0.08	ρ = −0.28	ρ = −0.33	ρ = −0.34	ρ = 0.1	ρ = −0.31	ρ = −0.33	ρ = 0.05
*p*-value	0.08	0.64	0.13	0.06	0.06	0.57	0.08	0.07	0.77
LDL-c	**ρ = 0.4**	r = 0.09	**ρ = 0.97**	r = 0.27	ρ = 0.3	r = 0.008	**ρ = 0.36**	r = 0.24	r = 0.19
*p*-value	**0.03**	0.62	**<0.01**	0.13	0.07	0.96	**0.05**	0.19	0.3
Total cholesterol	r = 0.33	r = 0.03	ρ = 0.33	r = 0.18	ρ = 0.26	r = 0.02	ρ = 0.29	r = 0.14	r = 0.23
*p*-value	0.07	0.83	0.07	0.33	0.15	0.91	0.11	0.43	0.21
Triglycerides	**ρ = 0.48**	ρ = 0.08	**ρ = 0.5**	ρ = 0.29	**ρ = 0.3**	ρ = 0.05	**ρ = 0.47**	ρ = 0.26	ρ = 0.19
*p*-value	**<0.01**	0.65	**<0.01**	0.11	**0.04**	0.75	**<0.01**	0.16	0.29
non-HDL-c	**ρ = 0.38**	r = 0.06	**ρ = 0.38**	r = 0.24	ρ = 0.3	r = −0.005	**ρ = 0.38**	r = 0.2	r = 0.22
*p*-value	**0.03**	0.71	**0.03**	0.19	0.1	0.97	**0.04**	0.26	0.23
TG/HDL-c ratio	**ρ = 0.46**	ρ = 0.14	**ρ = 0.46**	ρ = 0.31	**ρ = 0.38**	ρ = 0.002	**ρ = 0.45**	ρ = 0.31	ρ = 0.13
*p*-value	**0.01**	0.43	**<0.01**	0.08	**0.03**	0.98	**0.01**	0.08	0.47
TC/HDL-c ratio	**ρ = 0.39**	ρ = 0.08	**ρ = 0.37**	ρ = 0.33	ρ = 0.34	ρ = −0.05	**ρ = 0.36**	ρ = 0.30	ρ = 0.07
*p*-value	**0.03**	0.64	**0.049**	0.06	0.059	0.78	**0.04**	0.1	0.67
Fasting glucose	ρ = 0.26	r = 0.13	ρ = 0.25	r = 0.26	ρ = 0.37	r = 0.009	ρ = 0.28	r = 0.26	r = 0.19
*p*-value	0.16	0.46	0.17	0.16	0.04	0.95	0.12	0.16	0.31
GPT	**ρ = 0.44**	ρ = 0.35	**ρ = 0.41**	**ρ = 0.5**	**ρ = 0.46**	ρ = 0.15	ρ = 0.35	**ρ = 0.49**	ρ = 0.03
*p*-value	**0.01**	0.056	**0.02**	**0.04**	**0.01**	0.41	0.054	**<0.01**	0.85
GOT	**ρ = 0.5**	r = 0.23	**ρ = 0.48**	**r = 0.38**	**ρ = 0.46**	r = −0.07	**ρ = 0.44**	**r = 0.37**	r = 0.26
*p*-value	**<0.01**	0.2	**<0.01**	**0.03**	**<0.01**	0.7	**0.01**	**0.04**	0.16
Cortisol 8 am	ρ = 0.32	r = 0.031	ρ = 0.28	r = 0.1	ρ = 0.16	r = −0.002	ρ = 0.23	r = 0.07	r = 0.09
*p*-value	0.08	0.86	0.12	0.59	0.39	0.98	0.22	0.68	0.6
TSH	ρ = 0.06	r = 0.1	ρ = 0.06	r = 0.03	ρ = 0.09	r = −0.03	ρ = 0.21	r = 0.04	r = 0.21
*p*-value	0.74	0.58	0.73	0.87	0.61	0.86	0.25	0.83	0.25
25-OH- Vitamin D	**ρ = −0.45**	ρ = 0.02	**ρ = −0.42**	ρ = −0.3	**ρ = −0.36**	ρ = −0.05	ρ = −0.31	ρ = −0.29	ρ = −0.07
*p*-value	**0.01**	0.89	**0.01**	0.09	**0.05**	0.77	0.09	0.11	0.71
Ionized Calcium	**ρ = −0.59**	r = 0.04	**ρ = −0.44**	**r = −0.52**	**ρ = −0.49**	r = −0.17	**ρ = −0.38**	**r = −0.44**	r = 0.006
*p*-value	**<0.01**	0.8	**0.01**	**<0.01**	**<0.01**	0.36	**0.03**	**0.01**	0.97

Statistically significant results are indicated in bold.

**Table 11:** Correlations between arterial stiffness parameters and blood parameters in normal-weight children.

Correlations for Controls	PWV	AIx	SBP	DBP	MAP	HR	cSBP	cDBP	cPP
HDL-c	**r = −0.42**	**r = −0.47**	r = −0.35	ρ = −0.25	ρ = −0.25	r = −0.34	**r = −0.40**	**r = −0.43**	ρ = −0.22
*p*-value	**0.03**	**0.01**	0.08	0.21	0.22	0.09	**0.04**	**0.02**	0.27
LDL-c	r = 0.05	r = 0.07	r = 0.31	ρ = 0.05	ρ = 0.13	r = 0.03	r = 0.34	r = 0.29	ρ = −0.01
*p*-value	0.79	0.71	0.13	0.78	0.53	0.89	0.08	0.14	0.95
Totalcholesterol	r = −0.04	r = −0.05	r = 0.29	ρ = 0.03	ρ = 0.12	r = −0.002	r = 0.28	r = 0.23	ρ = −0.08
*p*-value	0.84	0.79	0.14	0.87	0.56	0.98	0.16	0.25	0.67
Triglycerides	ρ = 0.28	ρ = 0.37	**ρ = 0.4**	ρ = 0.3	ρ = 0.36	ρ = 0.15	**ρ = 0.4**	ρ = 0.29	ρ = 0.04
*p*-value	0.16	0.06	**0.04**	0.14	0.07	0.46	**0.05**	0.15	0.85
non-HDL-c	r = 0.11	r = 0.11	**r = 0.41**	ρ = 0.13	ρ = 0.24	r = 0.12	**r = 0.41**	r = 0.37	ρ = 0.02
*p*-value	0.58	0.57	**0.04**	0.51	0.24	0.56	**0.03**	0.06	0.91
TG/HDL-c ratio	ρ = 0.3	**ρ = 0.4**	**ρ = 0.4**	ρ = 0.36	**ρ = 0.39**	ρ = 0.17	ρ = 0.38	**ρ = 0.39**	ρ = 0.08
*p*-value	0.14	**0.04**	**0.04**	0.07	**0.05**	0.4	0.06	**0.05**	0.68
TC/HDL-c ratio	ρ = 0.38	ρ = 0.36	**ρ = 0.52**	**ρ = 0.41**	**ρ = 0.49**	ρ = 0.31	**ρ = 0.5**	**ρ = 0.54**	ρ = 0.11
*p*-value	0.06	0.07	**<0.01**	**0.04**	**0.01**	0.12	**<0.01**	**<0.01**	0.57
Fasting glucose	r = 0.04	r = −0.09	r = 0.02	ρ = 0.002	ρ = −0.06	r = −0.03	r = 0.02	r = −0.12	ρ = 0.1
*p*-value	0.85	0.64	0.9	0.99	0.76	0.87	0.91	0.55	0.61
GPT	**ρ = 0.48**	**ρ = 0.57**	ρ = 0.37	**ρ = 0.39**	ρ = 0.36	**ρ = 0.42**	ρ = 0.24	ρ = 0.24	ρ = 0.36
*p*-value	**0.01**	**<0.01**	0.06	**0.05**	0.07	**0.03**	0.24	0.24	0.07
GOT	ρ = 0.37	ρ = 0.3	ρ = 0.27	ρ = 0.15	ρ = 0.19	ρ = 0.13	ρ = 0.23	ρ = 0.09	**ρ = 0.44**
*p*-value	0.06	0.14	0.18	0.44	0.36	0.52	0.26	0.66	**0.02**
Cortisol 8 am	ρ = 0.23	ρ = 0.38	ρ = 0.24	ρ = 0.34	ρ = 0.29	ρ = 0.23	ρ = 0.25	ρ = 0.27	ρ = 0.007
*p*-value	0.25	0.056	0.24	0.09	0.15	0.25	0.23	0.18	0.97
TSH	r = −0.06	r = 0.1	r = −0.16	ρ = 0.1	ρ = −0.008	r = 0.17	r = 0.38	r = −0.23	ρ = −0.02
*p*-value	0.74	0.62	0.41	0.62	0.96	0.41	0.057	0.24	0.9
25-OH- Vitamin D	ρ = −0.12	ρ = −0.28	ρ = 0.11	ρ = 0.08	ρ = 0.09	ρ = −0.03	ρ = 0.1	ρ = 0.09	ρ = 0.02
*p*-value	0.56	0.16	0.58	0.69	0.63	0.86	0.61	0.65	0.89
Ionized Calcium	ρ = 0.33	ρ = 0.05	**ρ = 0.5**	ρ = 0.39	ρ = 0.51	ρ = 0.18	**ρ = 0.54**	**ρ = 0.53**	ρ = 0.39
*p*-value	0.1	0.79	**0.01**	0.053	0.008	0.38	**<0.01**	**<0.01**	0.048

Statistically significant results are indicated in bold.

**Table 12:** Multilinear regression for blood parameter predictors of PWA parameters.

Independent Variables	Dependent Variable (Significant)	Coefficient of Determination R^2^	Coefficient	Std. Error	t	*p*-Value
HDL-c	AIx	0.09	−0.33	0.14	−2.30	**0.02**
	DBP	0.26	−0.27	0.11	−2.34	0.02
LDL-c	PWV	0.19	0.006	0.001	3.55	**<0.01**
	SBP	0.22	0.15	0.03	3.90	**<0.01**
	cSBP	0.24	0.18	0.04	4.19	**<0.01**
	cDBP	0.32	0.08	0.03	2.52	0.01
Triglycerides	cPP	0.24	0.06	0.02	3.06	**<0.01**
TG/HDL-c ratio	DBP	0.26	2.17	0.71	3.07	**<0.01**
	HR	0.14	2.29	0.75	3.03	**<0.01**
	cPP	0.24	−2.49	0.7	−3.54	**<0.01**
TC/HDL-c ratio	MAP	0.25	3.50	0.81	4.28	**<0.01**
	cDBP	0.32	3.20	0.91	3.49	**<0.01**
GOT	AIx	0.34	−0.81	0.24	−3.27	**<0.01**
	MAP	0.17	0.21	0.09	2.38	0.02
	cSBP	0.35	0.57	0.1	5.45	<0.01
	cPP	0.15	0.25	0.08	3.09	**<0.01**
GPT	PWV	0.42	0.01	0.003	6.19	**<0.01**
	AIx	0.34	0.89	0.19	4.51	**<0.01**
	SBP	0.35	0.4	0.07	5.44	**<0.01**
	DBP	0.16	0.22	0.07	3.22	**<0.01**
	cDBP	0.30	0.34	0.07	4.85	**<0.01**
25-OH-Vitamin D	MAP	0.17	0.27	0.09	2.88	**<0.01**

Statistically significant results are indicated in bold.

## Data Availability

The data presented in this study are available only on request from the corresponding author. The data are not publicly available due to privacy restrictions.

## Abbreviations

AIx	index of augmentation
BMI	body mass index
BP	blood pressure
cDBP	central diastolic blood pressure
CI	interval of confidence
CIMT	carotid intima-media thickness
cPP	central pulse pressure
cSBP	central systolic blood pressure
DBP	diastolic blood pressure
GOT	aspartate aminotransferase
GPT	alanine aminotransferase
H	height
HDL-c	high-density lipoprotein cholesterol
HOMA-IR	homeostatic model assessment for insulin resistance
HR	heart rate
iCa	ionized Calcium
IFG	impaired fasting glucose
LDL-c	low-density lipoprotein cholesterol
MAP	mean arterial pressure
MetS	metabolic syndrome
*n*	number of subjects
NAFLD	non-alcoholic fatty liver disease
N-weight	normal-weight
PCOS	polycystic ovary syndrome
PWV	pulse wave velocity
PP	pulse pressure
SBP	systolic blood pressure
TC	total cholesterol
TG	triglycerides
W	weight
WC	waist circumference
